# Customized Scaffold Design Based on Natural Peripheral Nerve Fascicle Characteristics for Biofabrication in Tissue Regeneration

**DOI:** 10.1155/2019/3845780

**Published:** 2019-12-14

**Authors:** Zhi Yao, Li-Wei Yan, Shuai Qiu, Fu-Lin He, Fan-Bin Gu, Xiao-Lin Liu, Jian Qi, Qing-Tang Zhu

**Affiliations:** ^1^Department of Microsurgery and Orthopedic Trauma, First Affiliated Hospital of Sun Yat-sen University, Guangzhou, Guangdong Province, China; ^2^Center for Peripheral Nerve Tissue Engineering and Technology Research, Guangzhou, Guangdong Province, China; ^3^Guangdong Province Engineering Laboratory for Soft Tissue Biofabrication, Guangzhou, Guangdong Province, China

## Abstract

**Objective:**

The use of a biofabrication nerve scaffold, which mimics the nerve microstructure, as an alternative for autologous nerve transplantation is a promising strategy for treating peripheral nerve defects. This study aimed to design a customized biofabrication scaffold model with the characteristics of human peripheral nerve fascicles.

**Methods:**

We used Micro-MRI technique to obtain different nerve fascicles. A full-length 28 cm tibial nerve specimen was obtained and was divided into 14 two-centimetre nerve segments. 3D models of the nerve fascicles were obtained by three-dimensional reconstruction after image segmentation. The central line of the nerve fascicles was fitted, and the aggregation of nerve fascicles was analysed quantitatively. The nerve scaffold was designed by simulating the clinical nerve defect and extracting information from the acquired nerve fascicle data; the scaffold design was displayed by 3D printing to verify the accuracy of the model.

**Result:**

The microstructure of the sciatic nerve, tibial nerve, and common peroneal nerve in the nerve fascicles could be obtained by three-dimensional reconstruction. The number of cross fusions of tibial nerve fascicles from proximal end to distal end decreased gradually. By designing the nerve graft in accordance with the microstructure of the nerve fascicles, the 3D printed model demonstrated that the two ends of the nerve defect can be well matched.

**Conclusion:**

The microstructure of the nerve fascicles is complicated and changeable, and the spatial position of each nerve fascicle and the long segment of the nerve fascicle aggregation show great changes at different levels. Under the premise of the stability of the existing imaging techniques, a large number of scanning nerve samples can be used to set up a three-dimensional database of the peripheral nerve fascicle microstructure, integrating the gross imaging information, and provide a template for the design of the downstream nerve graft model.

## 1. Introduction

The clinical repair of peripheral nerve injuries is a difficult problem for patients and clinicians [1]. The number of serious injury cases in modern society is increasing. When evaluating different injury conditions, most clinical experts believe that nerve defects of degree III or above require nerve transplantation or bridging with a nerve repair material in order to repair and reconstruct normal function [2]. Clinicians continue to study the issue from various angles to identify more suitable clinical treatments [3], better transplantation materials [4], more stable timing of surgery [5], and so on. At present, autogenous nerve transplantation is still the “golden standard” for clinical treatment [6]. However, autogenous nerve transplantation is far from being satisfactory for clinicians. Its shortcomings are obvious: the source is extremely limited, it is difficult to match the nerve that needs to be repaired, the repair effect cannot be guaranteed, and a series of complications may occur in the donor area [7]. Therefore, autogenous nerve transplantation is not an ideal material for nerve transplantation [8].

As a result, many of the research teams are focusing on developing a nerve repair material that can be used to replace autografts. The existing commercial repair materials for the treatment of peripheral nerve defects mainly include two types: the nerve conduit [9] and the acellular nerve graft [10]. In their actual use, however, based on the experience of the clinical doctors, the nerve conduit is relatively simple in structure, far from the complex structure of the human nerve, and while a protective nerve conduit can be used for bridging a small section of a nerve defect, its effect in the repair of a long-section defect and for coarse nerves is poor [11]. The largest advantage of the acellular nerve repair material lies in its special natural three-dimensional structure and abundant natural extracellular matrix [12], and its repair effect is superior to that of the nerve conduit. However, the repair effect of the acellular nerve is still limited [13–15].

In the previous work of our research team on acellular nerves and its related basic experiments and development of the product [16, 17], we recognized that there is an important correlation between the biomimetic structure of acellular nerves and regeneration of the peripheral nerve axons [18]. Our team developed a rat sciatic nerve defect as an animal model and we found that changing the position of the autogenous nerve to match the nerve fascicles was beneficial to reduce the tortuosity and loss of nerve fibres in the regeneration process, and it improved the efficiency and quality of the nerve repair. The current experimental study proves that the physical guidance of the fascicle structure is vital in nerve regeneration [19]. Our experience coincides with the latest ideas of another research team, Zhu et al., who reported that 3D printing customized nerve grafts matching the structure of the sciatic nerve have shown good results in animal experiments [20]. The future application of customized nerve grafts in the treatment of complex peripheral nerve defects is worth exploring.

Therefore, what is the ideal nerve graft? Tissue-engineered nerve grafts have attracted much attention in recent years for producing biomimetic materials [21–23]. Clinical experts, materials experts, and engineering experts have been continuously exploring the biological mechanism of nerve regeneration from different angles [24]. It should be considered that the ideal peripheral nerve graft should have good physical strength, stability, biodegradability, safety, nontoxic chemical properties, and a biomimetic nerve microstructure and microenvironment [25]. However, the lack of understanding of the microstructure of the peripheral nerve and the roughness of the biofabrication methods [26, 27] are two difficult problems and bottlenecks in the preparation of new generation tissue-engineered nerve grafts.

Our team has established a technique of using Micro-MRI to recognize the internal microstructure of a peripheral nerve [28]. After three-dimensional reconstruction, in the Micro-MRI scan images, the morphology of the nerve fascicles and the cross fusion rules are clearly shown. The method has the advantages of being simple and convenient to perform, and the scanned sample can be recycled. Micro-MRI scanning technology provides a methodological basis for revealing the microstructure and the design of the biofabrication nerve graft model. In this study, the microstructure data from peripheral nerve fascicles in different nerves were collected by using the Micro-MRI imaging technique, and changes of the hierarchical structure of nerve fascicles were analysed quantitatively to expand the current knowledge of peripheral nerve microstructure. Understanding the complex morphological changes of the nerve fascicles will help us to understand the importance of matching the nerve microstructure in the design of the nerve graft. Furthermore, by simulating the clinical nerve defect, we extracted the necessary information from the obtained nerve fascicle data to design nerve grafts for the next generation of tissue engineering biomimetic nerve grafts for preliminary exploration and technical reserve.

## 2. Materials and Methods

### 2.1. Human Peripheral Nerve Samples

In accordance with the Declaration of Helsinki, all human experiments followed the procedures approved by the Institutional Review Committee of the participating institutions of the First Affiliated Hospital of Sun Yat-sen University. In this study, we used human nerves taken from amputated limbs of patients with bone tumours treated at the First Affiliated Hospital of Sun Yat-sen University. The selected collected peripheral nerve specimens had no tumour infiltration, no serious tissue defects or contamination, no thrombus formation in the lower limb aorta, and no obvious ischaemic signs of the limb before the amputation. The patients' nutritional states were good or moderate (no significant cachexia in the patient). All donors signed informed consent forms and agreed to donate their neural tissue for teaching and research purposes.

A 2 cm segment of the sciatic nerve, the tibia nerve, and common peroneal nerve from one patient were removed approximately 2 hours after the arterial ligation of the limb to be amputated. A total of 28 cm of the tibial nerve at the bifurcation from the sciatic nerve of the lower limb was cut into a total of 14 nerve samples 2 cm long. They were carefully trimmed under the microscope to cut off any adipose tissue around the nerve.

### 2.2. Different Peripheral Nerve Micro-MRI Scanning and Three-Dimensional Reconstruction of Nerve Fascicles

The main nerves of the lower limb were obtained: fresh samples of the sciatic nerve, tibial nerve, and common peroneal nerve were cut into 2 cm segments. The Micro-MRI scanning method was performed according to our previous work. A 0.1 mL aliquot of GD-DTPA (Bayer Healthcare, Berlin, Germany) was dissolved in 50 mL of iodine solution to give a 0.2% solution of the contrast agent. The nerve sample was placed at the bottom of a scan tube and the tube was sealed for scanning after filling it with the contrast agent and removing the air bubbles from the tube. After the preparation of the sample, the scan tube was placed in the head coil of the Micro-MRI (M3; Aspect Imaging, Jerusalem, Israel). The scan parameters are shown in Table 1.

The scanning process first performs nerve positioning according to the scout view and then starts the image scanning. The Micro-MRI image sequence is scanned to be introduced into the three-dimensional reconstruction software (Mimics Research 19.0; Materialise, Brussels, Belgium) with the selected grey value threshold range, and the mask is created. Then, the nerve fascicle boundary is artificially modified, a single nerve fascicle area mask is obtained by the segmentation template, a region growth algorithm is carried out according to the mask segmented in the continuous scanning image, and the three-dimensional reconstruction can obtain a three-dimensional model of each nerve fascicle.

### 2.3. Analysis of Long Segment Tibial Tract Cross Fusion

The tibial nerve of one lower limb with a full-length 28 cm from the bifurcation of the sciatic nerve from the malleolus was removed and divided into 14 two-centimetre segments of nerve samples. According to the above methods, Mimics Research 19.0 was used to reconstruct each segment of the nerve fascicles, and the 14 segments of the nerve fascicles were reconstructed and a three-dimensional model was obtained.

According to the three-dimensional reconstruction model of the nerve fascicles, the central line of the nerve fascicles was automatically fitted (Mimics Research 19.0: Analyze ⟶ Centerline ⟶ Fit centerline, Smooth factor: 0.5) and the intersection point of the central line was used to judge the cross fusion of the nerve fascicles here. The number of cross fusions of 14 segments of nerve fascicles was counted, and the number of cross fusions of the tibial nerve were analysed quantitatively.

### 2.4. Design of a Nerve Graft Model with a Simulated Nerve Defect in Accordance with the Microstructure of Nerve Fascicles

The three-dimensional reconstruction model of the tibial nerve bundle and nerve trunk was extracted from the above data and imported into 3-matic Research 11.0 (Materialise, Brussels, Belgium) to simulate the nerve defect. The middle segment of the nerve trunk was cut off to simulate the nerve defect, and the information about the tibial nerve fascicles was fitted to match the structure of the nerve fascicles at both ends of the defect. The main points of the design include neural gross morphology matching, neural branch matching, and three-dimensional spatial matching of the nerve fascicles. In this step, artificial fitting and matching are needed. The shape, curvature, and smoothness of the nerve trunk and nerve fascicles in the nerve graft are modified by three-dimensional model software. The nerve graft model was then obtained. After the mathematical model was obtained, the nerve graft design was displayed by 3D printing. The 3D printer used in the experiment is a single nozzle melt printer, and the nozzle diameter is 0.4 mm, print material is PLA, and printing temperature is 215°C.

## 3. Results

### 3.1. Micro-MRI Scanning of the Sciatic, Tibial, and Common Peroneal Nerves and Three-Dimensional Reconstruction of the Nerve Fascicles

**Figure 1:**
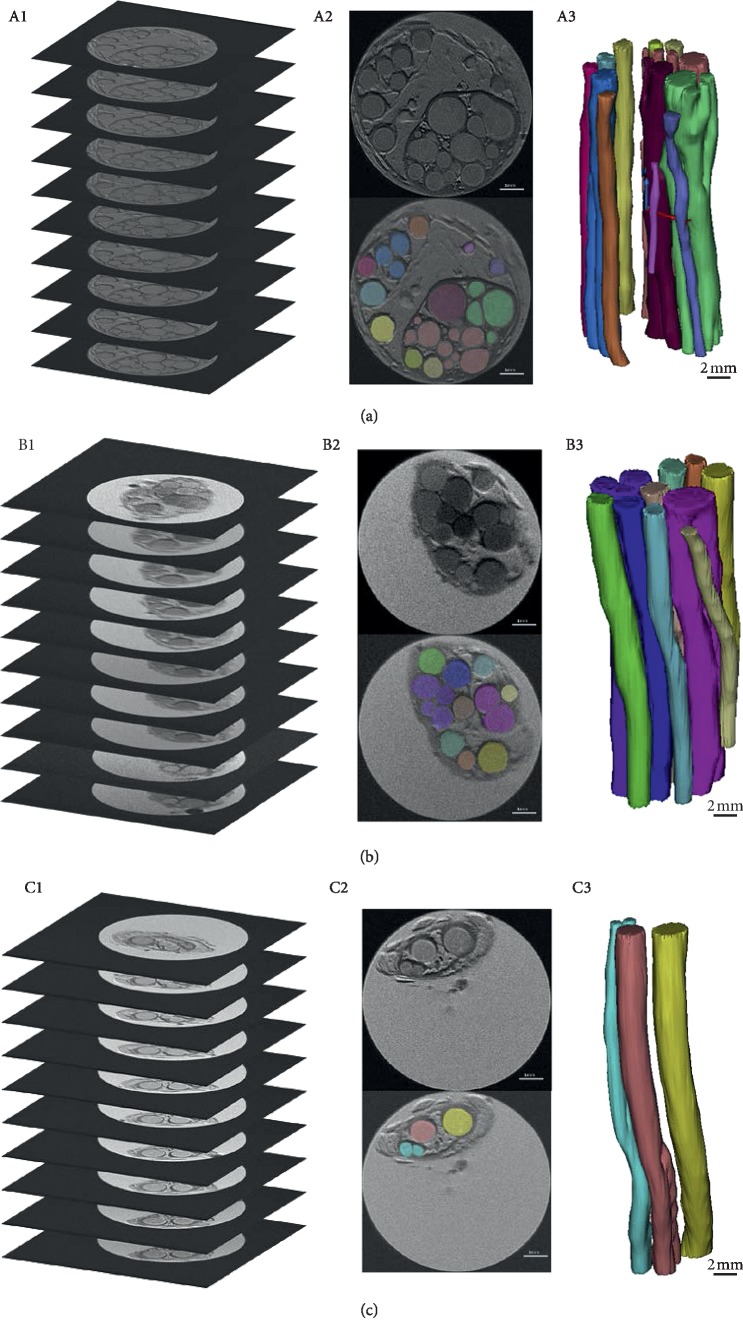
Micro-MRI scanning of the sciatic, tibial, and common peroneal nerves and three-dimensional reconstruction of the nerve fascicles. (a) Sciatic nerve Micro-MRI scan image (A1), two-dimensional image segmentation (A2), and three-dimensional reconstruction of nerve fascicles (A3). (b) Tibial nerve Micro-MRI scan image (B1), two-dimensional image segmentation (B2), and three-dimensional reconstruction of nerve fascicles (B3). (c) Common peroneal nerve Micro-MRI scan image (C1), two-dimensional image segmentation (C2), and three-dimensional reconstruction of nerve fascicles (C3). A2, B2, and C2: scale bar 1 mm; A3, B3, and C3: scale bar 2 mm.

The microstructure of nerve fascicles of the sciatic nerve, tibial nerve, and common peroneal nerve can be obtained by three-dimensional reconstruction.  Figure 1 shows the morphological differences of three-dimensional reconstruction of the nerves by Micro-MRI and nerve fascicles reconstruction. These are the original data for the later nerve graft design.

### 3.2. Analysis of Morphological Cross Fusion of the Long Tibial Nerve Bundle

**Figure 2:**
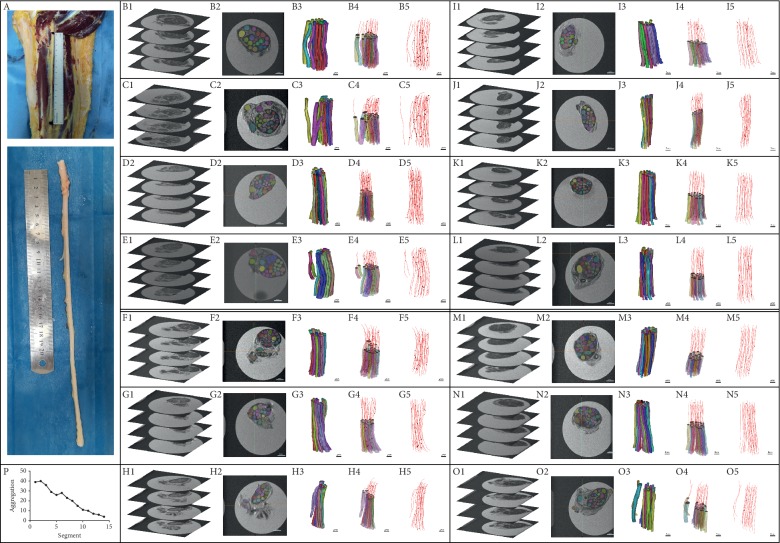
Analysis of morphological cross fusion of long tibial nerve fascicles. A: tibial nerve full-length specimen intercepted. B to O images showed 14 two-centimetre nerve samples taken from proximal end to distal end. In each box from left to right (1–5): Micro-MRI scan images are displayed in each group. In the two-dimensional image, the nerve fascicles were segmented, the nerve fascicles were reconstructed in three dimensions, and the central line was fitted to the three-dimensional reconstruction model. P: the change of the tibial nerve from sciatic nerve branches to the popliteal fossa: cross fusion of the nerve fascicles decreased gradually from proximal to distal. B2 to O2: scale bar 1 mm; B3 to O3, B4 to O4, and B5 to O5: scale bar 2 mm.

Figure 2 shows the three-dimensional reconstruction of the total length of the tibial nerve and the three-dimensional reconstruction of the 14 segments of the nerve fascicles. The cross fusion of the nerve fascicles gradually decreases from the proximal end to the distal end. The number of cross fusions of nerve fascicles from the proximal end to the distal end in this study is 39, 40, 36, 32, 29, 26, 28, 23, 20, 15, 11, 10, 7, 6, and 4. The cross fusion of the nerve fascicles at the posterior tibial nerve of the sciatic nerve was the highest, and the number of cross fusions of the nerve fascicles gradually decreased as the tibial nerve continued to branch along the way.

### 3.3. Design of Nerve Graft in Accordance with the Morphological Structure of Nerve Fascicles by Simulating a Nerve Defect

**Figure 3:**
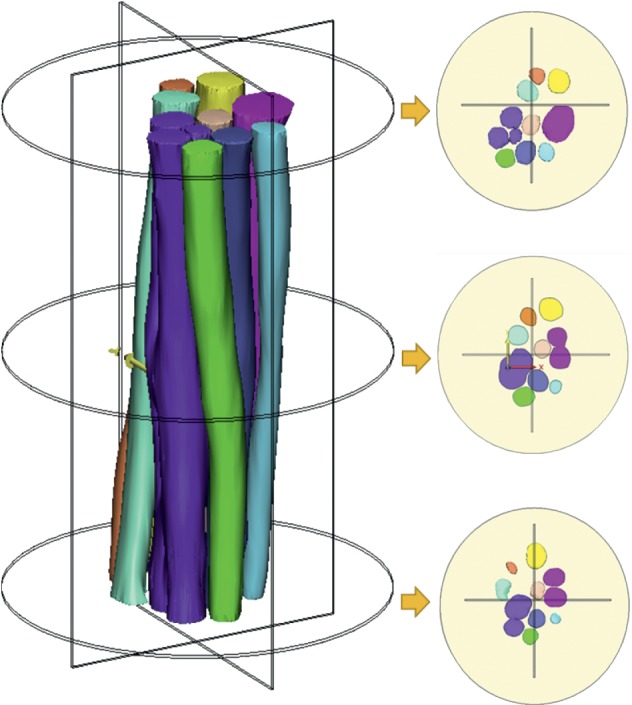
Spatial location and distribution change of nerve fascicles in the three-dimensional reconstruction: the distribution of nerve fascicles in the distant, near, and middle segments was intercepted, and the changes of the spatial position of the nerve bundle over a short distance can be observed.

**Figure 4:**
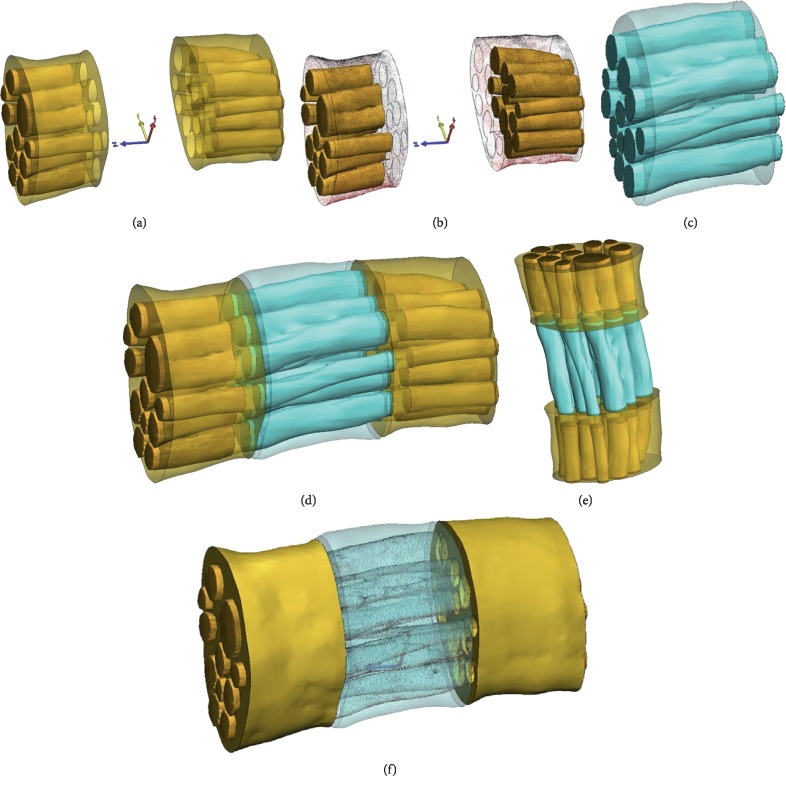
A biofabrication model that mimics the microstructure of peripheral nerve fasciculus was designed based on the three-dimensional database: In scenarios of patients with nerve defects, a nerve graft was designed and customized to the nerve defects. (a) Distal and proximal part of the nerve defect. (b) Differences in the fascicular structure of the distal and proximal part of the nerve defect. (c) The nerve graft was designed according to the three-dimensional database of nerve fasciculus. (d, e, and f) Simulation of the matching between the nerve graft and nerve defect.

**Figure 5:**
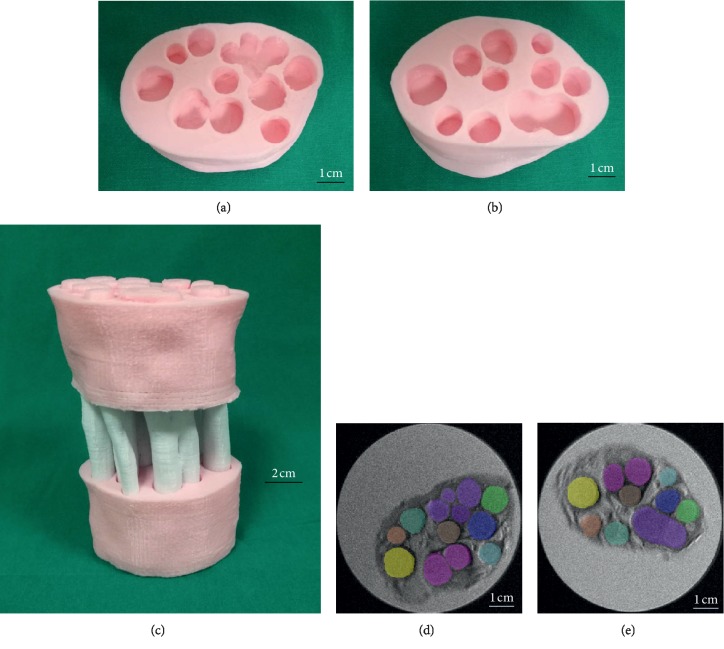
The 3D printing model demonstrates the morphological characteristics of the customized nerve graft that mimics the microstructure of the peripheral nerve fasciculus. (a, b) Significant differences in the number and spatial distribution of the nerve fasciculus in the proximal and distal part of the nerve defect. (c) The 3D printing PLA model demonstrates the morphological features of the customized nerve graft, showing the nerve graft and the proximal and distal parts of the nerve defect are well matched. (d, e) The distribution of the two nerve tracts corresponding to the original Micro-MRI scan image. (a, b) Scale bar 1 cm. (c) Scale bar 2 cm. (d, f) Scale bar 1 mm.

There are great differences in the morphological structures of nerve fascicles in different nerve specimens and in the same nerve specimen (Figure 3). With the deepening of the understanding of the microstructure, we realize the importance of guiding the morphological and physical structure of the nerve fascicles. Clinical 3T MRI may be used to scan the gross morphology of the nerve trunk on both the healthy side and the affected side, and then the nerve graft was designed according to the information extracted from the nerve bundle database according to the specific injury situation. Nerve graft design should have the following characteristics: nerve gross morphology matching, nerve branch matching, and nerve bundle three-dimensional space matching. Figures 4 and 5 show a biofabrication nerve graft model based on the three-dimensional reconstruction of the tibial nerve obtained earlier, which accords with the morphological characteristics of the nerve fascicles. The microstructures of the distal and proximal nerve fascicles of the peripheral nerve defect are different. The nerve graft designed by the tibial nerve can be matched with the two ends of the nerve defect. And the modified three-dimensional model has great similarity compared with the original nerve fascicles Micro-MRI scanning image after the 3D printing.

## 4. Discussion

The internal structure of the peripheral nerve and the morphology of the nerve fascicles are complicated [29]. It was proven in a previous study by our team that the nerve fascicles change in position and quantity within a distance of 0.25∼5 mm, and this is accompanied by the differentiation and combination of the functional nature of the nerve [30]. The understanding of peripheral nerves is limited at the technical level and the amount of data studied. With the continuous development of computer software and hardware in the last decade, the ability of three-dimensional reconstruction of medical images has developed rapidly. Based on the continuous tissue sections of peripheral nerves, the authors have tried to reconstruct the three-dimensional anatomy of these peripheral nerves more deeply. The stained continuous sections can be used to obtain more precise and accurate images of the nerve sections than those of their simple microanatomy [31]. However, this method still requires manual manipulation in image registration and contour acquisition, which not only increases the working intensity but also reduces the accuracy of three-dimensional reconstruction of neural functional fascicles [32].

With the continuous development of information technology, from traditional histology staining to modern imaging technology, 3D visualization and holographic imaging technology also make neuroscience research more systematic and more accurate. The new development of medical imaging technology, Micro-CT and Micro-MRI, has brought about a breakthrough in the study of nerve fascicles microstructure. The resolution of Micro-CT imaging is high [33]. The nerve can be pretreated with an iodine agent combined with a freeze-drying method and the obtained images can distinguish the anatomical structure to a resolution of less than 10 *μ*m. It can even be used to analyse the mechanical properties of tissue scaffolds, which can help to improve the design and manufacture of the anatomical structure of scaffolds [34]. While maintaining the overall small deformation rate of the specimen, two-dimensional images of the peripheral nerve can be obtained by high-precision scanning with a Micro-CT. Three-dimensional reconstruction and computer visualization technology can be used to observe the internal structure of the nerve and the shape of the nerve fascicles.

Our team previously introduced the Micro-MRI scanning technique of obtaining continuous scanning images of peripheral nerves and used these images to reconstruct the three-dimensional structure of the nerve fascicles [28]. The Micro-MRI technique is efficient and convenient. Its outstanding advantage is that the shape of the specimen remains stable during the scanning process and that the scanned nerve specimen can be recycled. However, current medical imaging technology is not perfect. It still faces many technical problems, such as image enhancement and optimization, image microdeformation and restoration, three-dimensional reconstruction optimization, massive two-dimensional image demand for hardware and software, and digital modelling and structure optimization. Therefore, it still needs more exploration and research to present the “real structure.”

According to the present research results, it has been found that the hierarchical morphological structure of the nerve fascicles is complex and changeable, and the spatial position of each nerve fascicle and the long segment of the nerve fascicles cross fusion in different nerves and different planes show great changes. The site, nature, and length of injury of patients with peripheral nerve defects are different, and the corresponding internal nerve fascicles structure is very different. It is obviously difficult to achieve a satisfactory curative effect by using universal repair materials.

**Figure 6:**
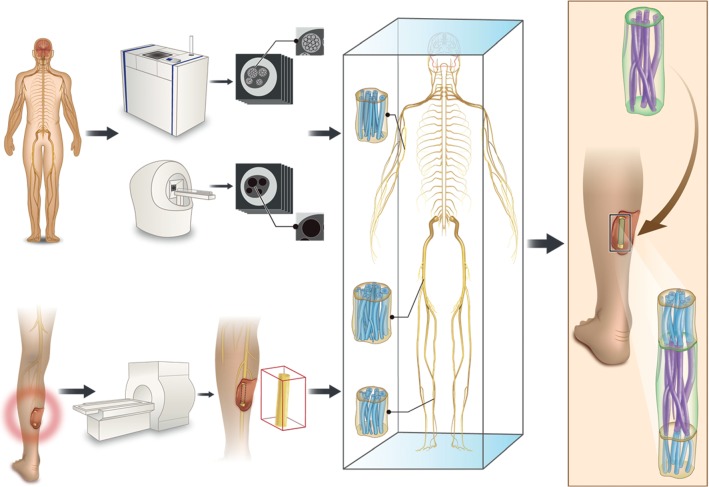
The establishment of the database of peripheral nerve microstructure and its application to design a biofabrication nerve graft model according to the morphological characteristics of the nerve bundle. The three-dimensional information database of the peripheral nerve fascicles microstructure was established by scanning peripheral nerve samples using a large number of imaging techniques (Micro-MRI/Micro-CT). When patients with peripheral nerve defects are encountered in the clinic, 3T MRI is used to scan the gross morphology of the nerve trunk of the healthy side and the affected side to locate the damaged area, the size of the defect, and whether the nerve branches were sent out. The model design information was extracted by matching from the database the gross size of the nerve branch and three-dimensional spatial information about the nerve bundle. A biofabrication nerve graft model of the nerve repair material was designed according to the microstructure of the nerve bundle according to the data from clinical MRI nerve trunk scanning.

On the basis of summarizing the existing neural microstructure data, our team proposed the design idea of the next generation of customized scaffolds based on natural peripheral nerve fascicles characteristics for the biofabrication in tissue regeneration (Figure 6). In this study, by simulating the clinical nerve defect design, we extracted the information from the obtained nerve fascicles data to design a biofabrication nerve graft model for the pretechnical reserve. Under the premise of a stable imaging technique (Micro-MRI/Micro-CT), a large number of scanning nerve samples can be used to set up a three-dimensional information database of peripheral nerve fascicle microstructures. It can provide a template for the design of the whole chain downstream nerve graft model. In the future, nerve graft design should reflect this microstructure information, design matching nerve fascicle microstructure characteristics of biomimetic repair nerve grafts, and achieve nerve gross morphology matching, nerve branch matching, and three-dimensional spatial pattern matching of nerve fascicles morphology. Tissue engineering customized nerve grafts can be a new solution to repair nerve defects in the future [35–37].

The limitations of this study are mainly due to the existing experimental conditions and the small human nerve sample size. The accuracy of neural fascicle cross fusion is affected by the accuracy of the three-dimensional reconstruction model. Therefore, the higher the accuracy of the original image is, the more accurately the 3D model can reflect the morphology of the original nerve fascicles. Under the condition of the existing 1T Micro-MRI equipment, the resolution of the scanning image is 50 *μ*m and thus the distance between different scanning planes is still large. The overall trend of the cross fusion of the tibial nerve full-length nerve fascicles is real. However, the specific nerve fascicle cross fusion statistical times still need a larger number of samples for reference.

To provide ideas for the design of the next-generation nerve graft, the design method of the biofabrication nerve graft model, which is in accordance with the characteristics of neural morphology and microstructure, has been put forward in this study. This study is an exploration of and technical reserve for individualized matching nerve graft design in the future. At present, it is impossible to fabricate nerve grafts with such complex structures by biofabrication means [38–40]. Several studies reported that 3D printing nerve grafts have shown good results in animal experiment; however, the 3D printed structure they present is still relatively simple. The main reason is limited to printer accuracy and physical strength and molding properties of printing bioink. The existing research conditions cannot realize 3D printing of small size with complex structure of soft tissue. Therefore, the next step in research for model design part should focus on how to simplify the hierarchical morphological information of the nerve fascicles and match them with the existing biological manufacturing methods. Thus, the exploration of ideal nerve repair materials is still necessary, and it needs long-term work to finally switch from a quantitative change to a qualitative change. Breakthroughs in related technologies require cross-disciplinary cooperation, such as clinical medicine, material science, computer science, and engineering.

## 5. Conclusion

The morphological structure of the nerve fascicles is complex and changeable, and the spatial position of each nerve fascicle in different planes and the cross fusion of the long nerve fascicles can show great changes. The Micro-MRI scanning technique can increase our understanding of the morphology of the nerve fascicles. The three-dimensional information database of nerve fascicle morphology provides a template for the design of customized scaffolds based on natural peripheral nerve fascicle characteristics for biofabrication for use in tissue regeneration.

## Figures and Tables

**Table 1:** Micro-MRI scan parameters.

	Parameter
Slice orientation	Axial
Number of slices	25
Slices thickness (mm)	1
Interslice gap (mm)	0.1
Hor. FOV (mm)	15
Vert. FOV (mm)	15
No of. phase encodings	300
No of. sample	300
Time to repeat (TR, ms)	607.025
Time to echo (ms)	15.834
Inversion time (TI, ms)	100
Pixel size (mm)	0.05
No of. excitations	50
Scan time	2 : 45 : 55

## Data Availability

The Micro-MRI scanning technique for peripheral nerve used to support the findings of this study has already been published (doi:10.4103/1673-5374.238718). The original Micro-MRI scanning images and 3D models used to support the findings of this study are available from the corresponding author upon request.
